# Microbial uptake kinetics of dissolved organic carbon (DOC) compound groups from river water and sediments

**DOI:** 10.1038/s41598-019-47749-6

**Published:** 2019-08-02

**Authors:** Francesca L. Brailsford, Helen C. Glanville, Peter N. Golyshin, Penny J. Johnes, Christopher A. Yates, Davey L. Jones

**Affiliations:** 1 Bangor University, Environment Centre Wales, Bangor, LL57 2UW UK; 2 Bangor University, Centre for Environmental Biotechnology, Bangor, LL57 2UW UK; 3 Keele University, School of Geography, Geology and the Environment, Keele, Newcastle-under-Lyme, ST5 5BG UK; 4 University of Bristol, School of Geographical Sciences, University Road, Bristol, BS8 1SS UK; 5 The University of Western Australia, School of Agriculture and Environment, Crawley, WA 6009 Australia

**Keywords:** Biogeochemistry, Biogeochemistry, Biogeochemistry, Biogeochemistry, Biogeochemistry

## Abstract

Dissolved organic matter (DOM) represents a key component of carbon (C) cycling in freshwater ecosystems. While the behaviour of bulk dissolved organic carbon (DOC) in aquatic ecosystems is well studied, comparatively little is known about the turnover of specific DOC compounds. The aim of this study was to investigate the persistence of ^14^C-labelled low molecular weight (LMW) DOC at a wide range of concentrations (0.1 µM to 10 mM), in sediments and waters from oligotrophic and mesotrophic rivers within the same catchment. Overall, rates of DOC loss varied between compound groups (amino acids > sugars = organic acids > phenolics). Sediment-based microbial communities contributed to higher DOC loss from river waters, which was attributed, in part, to its greater microbial biomass. At higher DOC compound concentrations, DOC loss was greater in mesotrophic rivers in comparison to oligotrophic headwaters. A lag-phase in substrate use within sediments provided evidence of microbial growth and adaptation, ascribed here to the lack of inorganic nutrient limitation on microbial C processing in mesotrophic communities. We conclude that the higher microbial biomass and available inorganic nutrients in sediments enables the rapid processing of LMW DOC, particularly during high C enrichment events and in N and P-rich mesotrophic environments.

## Introduction

Dissolved organic carbon (DOC) is a complex mixture of compounds and represents a key component of carbon (C) transfer from terrestrial to freshwater environments and from headwaters to the marine zone^1^. Further, allochthonous, terrestrially-derived DOC is frequently believed to be largely recalcitrant in freshwaters merely being transported rather than transformed in the aquatic environment. However, recently it has been shown to represent an important source of bioavailable carbon (C), fuelling aquatic heterotrophic ecosystem processes, particularly in streams and rivers influenced by peat-dominated headwaters where DOC concentrations are particularly high^2,3^. DOC compounds can influence a wide range of processes occurring in the aquatic environment^4^. For example, high molecular weight (MW) DOC compounds have been found to bind to extracellular enzymes, modulating DOC breakdown along an aquatic continuum^5^. The fact that a DOC gradient exists along the majority of rivers, which abiotic degradation alone cannot account for, indicates that biological processing of DOC in-stream is occurring^5–7^.

Sediments represent a crucial element of in-stream DOC processing due to the constant transfer of waters and nutrients occurring through the hyporheic and groundwater zone in catchments^8–10^. These hyporheic-zone interactions are thought to have a major control on the residence time of organic matter compounds in freshwaters^11^. Sediments can accumulate nutrients over time, particularly in lowland, low-gradient waters where sedimentation is more likely to occur^6^. Sediments can also be an autochthonous DOC source; it has been suggested that there is a net DOC efflux from sediments to overlying waters^12,13^. Sediments also have the potential to become a primary source of pollutants, such as heavy metals, to overlying waters if there is a change to the aquatic chemical properties, leading to benthic nutrient export^14^.

Aquatic ecosystems are subject to increasing pressures; over the last few decades there have been increases in DOC fluxes from uplands across Europe and North America, particularly those dominated by peats, likely due to increasing global temperatures or a change in atmospheric N and S deposition^15^. In addition, anthropogenic inputs of excess inorganic nutrients to rivers promotes microbial activity, leading to reduced oxygen availability, eutrophication and disruption of entire food chains and loss of ecosystem services^16–18^. In addition, it has been established that a small change in DOC concentrations can also lead to a shift in aquatic microbial community structure^19^. How microbial aquatic communities respond to changes in DOC inputs is not clear; consequently, this paper investigates the response of microbial communities to a range of DOC inputs.

While ultra-high resolution mass spectrometry has the potential to trace individual compounds through aquatic environments^20^, few studies have quantified the pool sizes and fluxes of individual DOC compounds in freshwaters. A review of methods for measuring the microbial processing of DOC in lentic waters indicated that a ^14^C-labelled DOC tracer approach can be employed to measure DOC processing by the microbial community^21^. The two main approaches are to either (1) add ^14^C-tracers at intrinsic concentrations and measure uptake from solution and subsequent ^14^CO_2_ respiration following metabolism, or (2) a kinetics approach measuring uptake at a wide range of concentrations, from concentrations below ambient conditions to high concentrations intended to fully saturation the system, in order to estimate rate parameters e.g. K_m_ and V_max_ for specific DOC compounds^21^.

To date, there have been a limited number of studies applying these methods to aquatic environments; such studies have focused on waters only, using simple ^14^C-labelled DOC compounds in isolation rather than compound groups, e.g. glucose or phenol^22,23^. However, DOC is a heterogeneous mixture of compounds. Therefore we advocate that taking a specific compound group approach (using multiple compounds added together) will provide a more representative estimate of DOC loss rates in aquatic environments. This approach has been taken in some soil-based studies where more complex groups of DOC compounds have been investigated, such as amino acids^24^ and organic acids^25^. The kinetics-based approach using a large range of concentrations of the same compound or compounds has also been conducted successfully in some soil-based studies, primarily for glucose and other simple sugars^26–28^. To our knowledge, there have been no previous studies using a kinetics-based approach for more complex phenolic compounds, which are a key component of the DOC pool in upland headwaters and sediments, accounting for up to 75% of the bioavailable DOC present^29,30^.

The aims of this study were therefore to: (1) compare the rates of microbial uptake of four groups of low molecular weight (LMW) DOC compounds over time (sugars, amino acids, organic acids and phenolics); (2) determine the ability of microbial populations to process DOC under differing catchment conditions; and (3) establish the role of sediment and the hyporheic zone on DOC processing in rivers. The results of the study will be used to evaluate the relative importance of water-column versus hyporheic zone driven DOC processing and to establish trends in preferential uptake of any DOC fractions between mesotrophic and oligotrophic rivers.

## Methods

### Field sites and sampling

Sediment and water samples were collected from two contrasting land cover types within the Conwy catchment, North Wales, UK^31^ (Fig. S1). The first set were collected from three independent mesotrophic streams passing through lowland improved grasslands (mainly Cambisol soil type with some Gleysols present and *Lolium perenne* L. and *Trifolium repens* L. dominated swards). These livestock (sheep and beef) grazed grasslands have a long history (>70 y) of receiving organic wastes in the form of cattle manures and slurries, inorganic NPK fertilisers and lime. The second set were collected from three independent oligotrophic headwater streams draining an upland blanket peat bog (mainly Histosol soil type) dominated by acid heathland vegetation (e.g. *Calluna vulgaris* (L.) Hull, *Vaccinium myrtillus* L., *Eriophorum vaginatum* L.), low intensity sheep grazing (<0.1 ewe ha^−1^) and no history of fertiliser application.

During the winter of 2016, three replicate 30 g samples of sediment (0–2 cm depth) were collected close to the riverbank at each site. In addition, three replicate unfiltered water samples were collected manually in acid-washed 1 L high density polyethylene (HDPE) bottles, 1 m upstream from the sediment sampling sites. Samples were placed in labelled bags and transported back to the laboratory at 10 °C in the dark within 4 hours of collection. pH and electroconductivity (EC) of river water and 1:2.5 (w/v) suspensions of sediment in e-pure water (18 MΩ resistance) were measured on the same day using standard electrode probes. Within 24 hours of collection, aliquots of river water, 1:5 sediment-to-1 M CH_3_COOH extracts for P analysis and 1:5 sediment-to-0.5 M K_2_SO_4_ for all other analyses were frozen at −20 °C until subsequent laboratory analysis.

### Background chemical analysis

Sediment moisture content was determined by oven drying <2 mm sieved sediment at 105 °C for 24 h. Organic matter content was measured using loss-on-ignition in a muffle furnace (450 °C, 16 h)^32^. Oven dried, root free sediment was analysed for C and N content using a TruSpec^®^ analyzer (Leco Corp., St Joseph, MI, USA). Sediment samples were collected and shipped to Yara (Lincolnshire, UK) for texture analysis (Sand %, Silt %, Clay %) using a Mastersizer 3000 laser particle size analyzer (Malvern Panalytical). River water DOC and total dissolved N (TDN) content were determined using Multi N/C 2100 S analyser (AnalytikJena, Jena, Germany). The following chemical parameters were determined using river water samples and 0.5 M K_2_SO_4_ sediment extracts: concentrations of NH_4_^+^ and NO_3_^−^ were measured according to the methods outlined by Mulvaney^33^ and Miranda^34^ respectively. Total free amino acids and total free carbohydrates were determined using the fluorometric OPAME procedure^35^ and the Myklestad method^36^ respectively. The concentration of phenolic compounds was measured using the Folin-Ciocalteu method^37^. Finally, molybdate-reactive P was measured for river water samples and 1 M 1.0 M CH_3_COOH sediment extracts^38^.

### Microbial community analysis

To determine the size and structure of the microbial community, phospholipid-derived fatty acid (PLFA) analyses were carried out on both river water and sediment samples. From each site a 25 L water sample was collected and concentrated in the laboratory to 50 mL using a KrosFlo Research IIi Tangential Flow Filtration System (Spectrum Laboratories Inc., Rancho Dominguez, CA, USA). Concentrated water samples and 25 g sediment samples were then freeze-dried and stored at −80 °C until shipping on dry ice to Microbial ID, DA, USA. The PLFA content of the samples was determined using the methods outlined by Buyer and Sasser^39^.

### DOC depletion experiment

Within 6 hours of collection, three independent replicate samples containing 9.9 mL unfiltered water and three replicates of 9.9 mL unfiltered water plus 1.00 g (±0.01) sediment were added to sterile 15 mL polypropylene centrifuge tubes (Corning Inc., Corning, NY, USA). The river-water only treatment represented the response of the water column microbial biomass to different DOC inputs, whereas the sediment and river water (1:10 ratio) treatment represented the interaction of the water column, groundwater zone and the sediment porewater microbial biomass within the hyporheic zone. Each sample then had 100 µL of solution containing unlabelled DOC compounds (at the final concentrations outlined below), spiked with the corresponding ^14^C-labelled compounds to act as a tracer, with a final activity 0.4 kBq mL^−1^. The amount of ^14^C-tracer added to each DOC solution was <1 nM and therefore not expected to change the overall concentration. The kinetic assays were divided into four compound groups: sugars, amino acids, organic acids and phenolic compounds. In total, 8 different radioisotopically labelled compounds were used (Table S1). Compounds were chosen to reflect compounds typically released during the breakdown of particulate organic matter entering soils and freshwaters. Sterile controls run with e-pure water (18 MΩ resistance) in place of river water resulted in no loss of ^14^C-compounds from solution (Fig. S2). Abiotic loss of ^14^C-compounds due to sorption was accounted for by running sediment controls sterilised with formaldehyde; these values were then used to correct for any potential sorption at high and low ^14^C-compound concentrations (Fig. S3).

The concentrations of amino acids (alanine, arginine, aspartate, glutamate, glycine, isoleucine, lysine, methionine, phenylalanine, proline, serine, tyrosine, valine), glucose, organic acids (acetic acid, citric acid, malic acid) and phenolic compounds (*P*-coumaric acid, salicylic acid, vanillic acid) ranged from 0.1 µM to 0.5 mM for waters and 0.1 µM to 10 mM for sediments (Table S2). A higher concentration range for sediments was utilised to represent the higher background DOC concentrations found in sediments. The wide range of concentrations were selected to represent a broad range of DOC conditions, from low ambient concentrations through to an excess of DOC capable of fully saturating the system, which although unlikely to occur naturally for a prolonged period were used to assess the maximum concentration that could be processed in the two contrasting systems. After sealing with sterile caps, the samples were subsequently incubated on a shaker (200 rev min^−1^) in the dark at 10 °C, to ensure the samples remained well-mixed and aerated for the duration of the experiment. This temperature represents the mean annual temperature within the catchment^23^. The mean water temperature over the duration of the experiment was 8.28 ± 0.34 °C.

**Figure 1:**
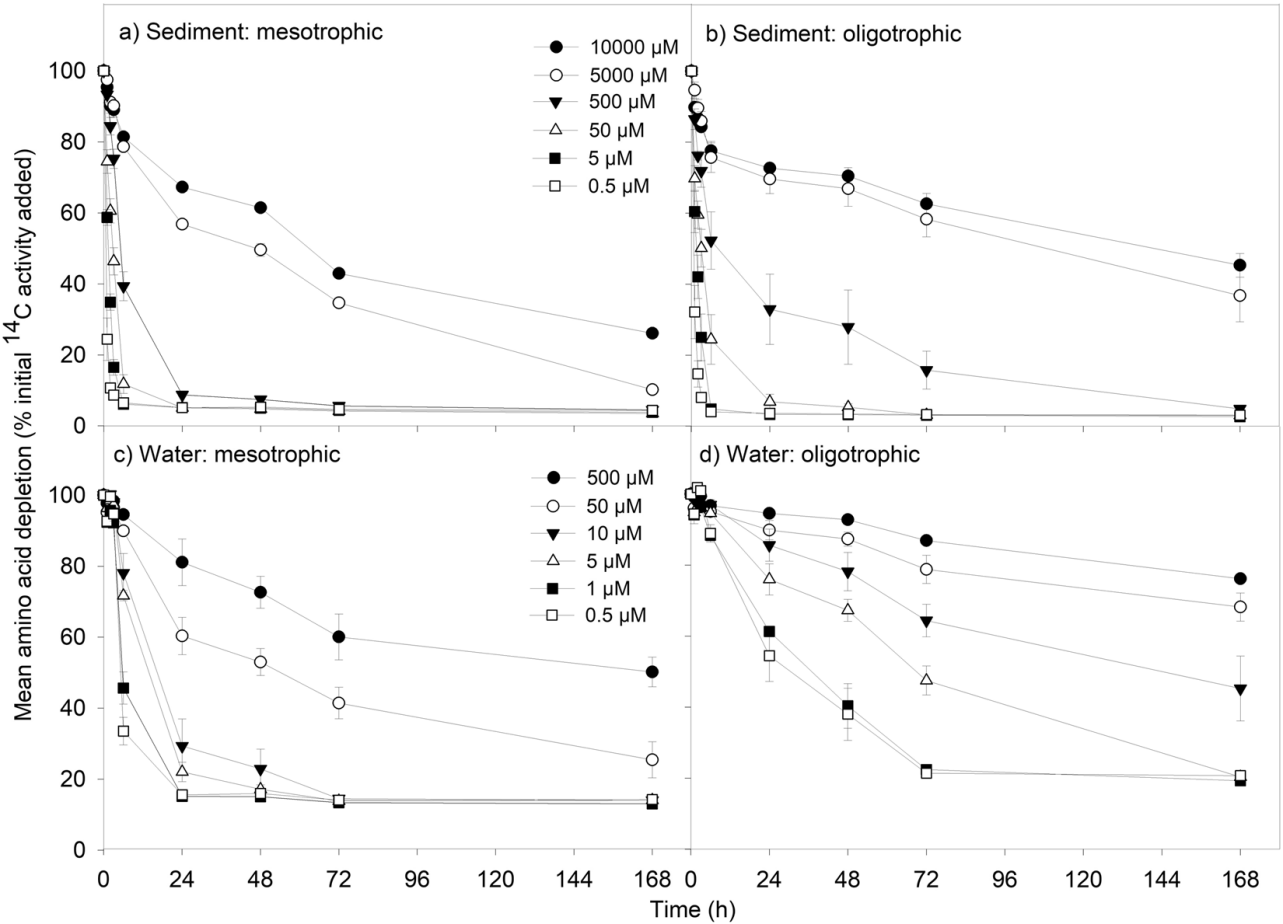
Effect of DOC concentration on the loss of ^14^C-labelled amino acids for: (**a**) lowland improved grassland river sediments (mesotrophic), (**b**) upland peat bog sediments (oligotrophic), (**c**) lowland improved grassland river waters (mesotrophic), (**d**) upland improved grassland river waters (oligotrophic). Values represent means ± SEM (*n* = 3). Please note the legends are different for the top two panels (a and b) and bottom two panels (c and d) to represent the different concentration ranges found in each substrate type; sediment and water respectively. The legend is the same for the top two (**a** and **b**) and bottom two (**c** and **d**) panels respectively.

**Figure 2:**
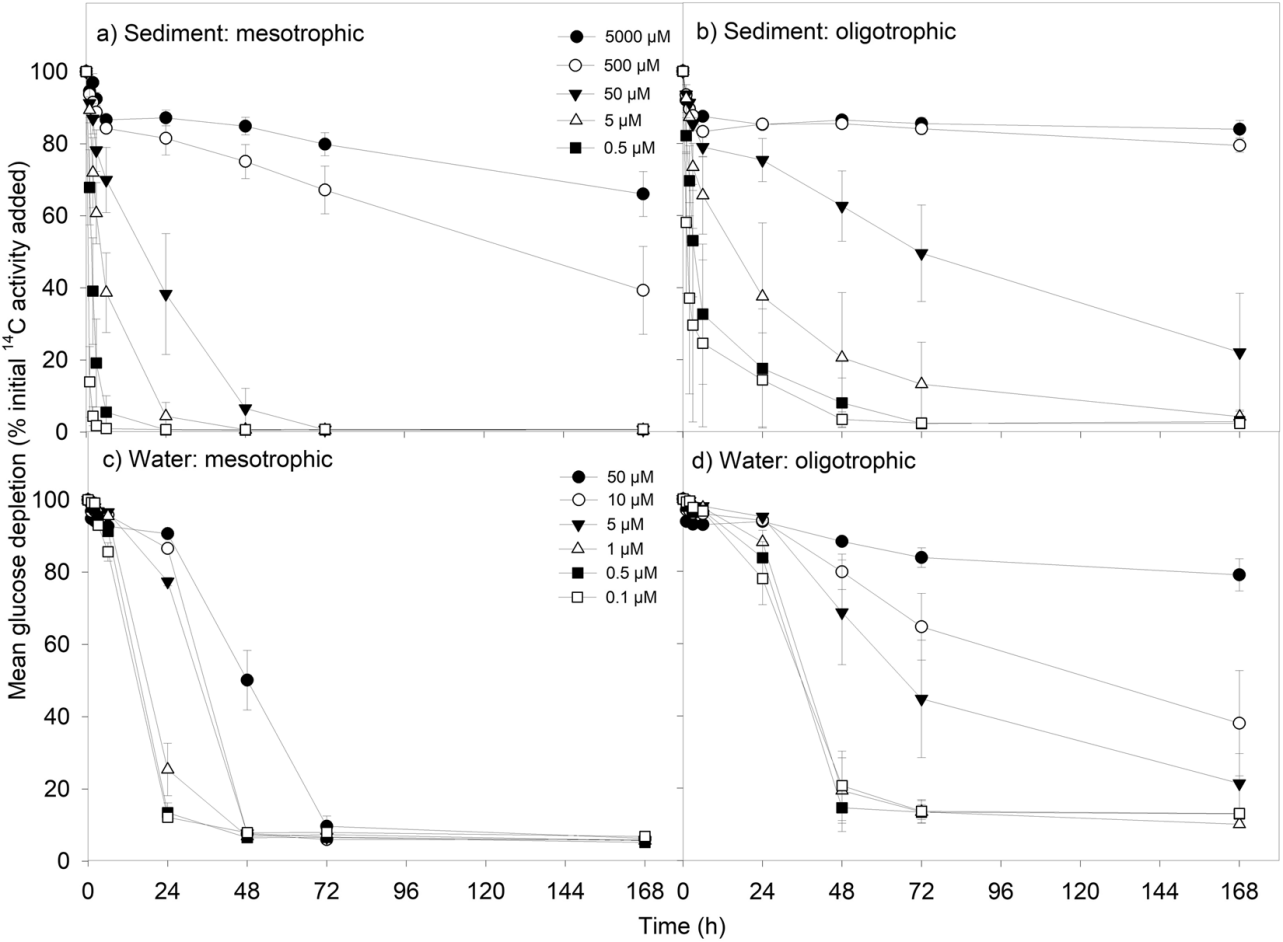
Effect of DOC concentration on the loss of ^14^C-labelled glucose for: (**a**) lowland improved grassland river sediments (mesotrophic), (**b**) upland peat bog sediments (oligotrophic), (**c**) lowland improved grassland river waters (mesotrophic), (**d**) upland improved grassland river waters (oligotrophic). Values represent means ± SEM (*n* = 3). Please note the legends are different for the top two panels (a and b) and bottom two panels (c and d) to represent the different concentration ranges found in each substrate type; sediment and water respectively. The legend is the same for the top two (**a** and **b**) and bottom two (**c** and **d**) panels respectively.

**Figure 3:**
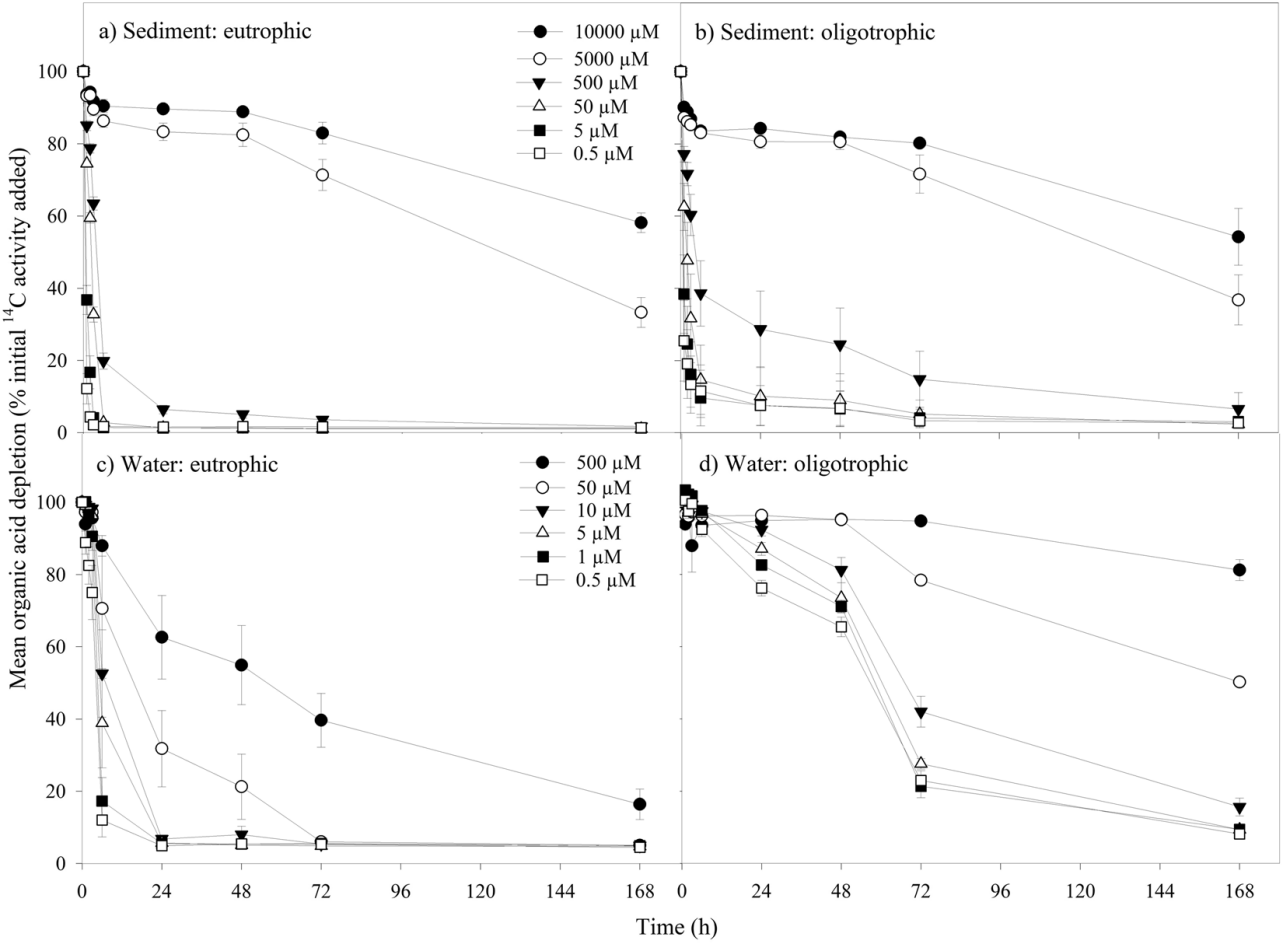
Effect of DOC concentration on the loss of ^14^C-labelled organic acids for: (**a**) lowland improved grassland river sediments (mesotrophic), (**b**) upland peat bog sediments (oligotrophic), (**c**) lowland improved grassland river waters (mesotrophic), (**d**) upland improved grassland river waters (oligotrophic). Values represent means ± SEM (*n* = 3). Please note the legends are different for the top two panels (a and b) and bottom two panels (c and d) to represent the different concentration ranges found in each substrate type; sediment and water respectively. The legend is the same for the top two (**a** and **b**) and bottom two (**c** and **d**) panels respectively.

**Figure 4:**
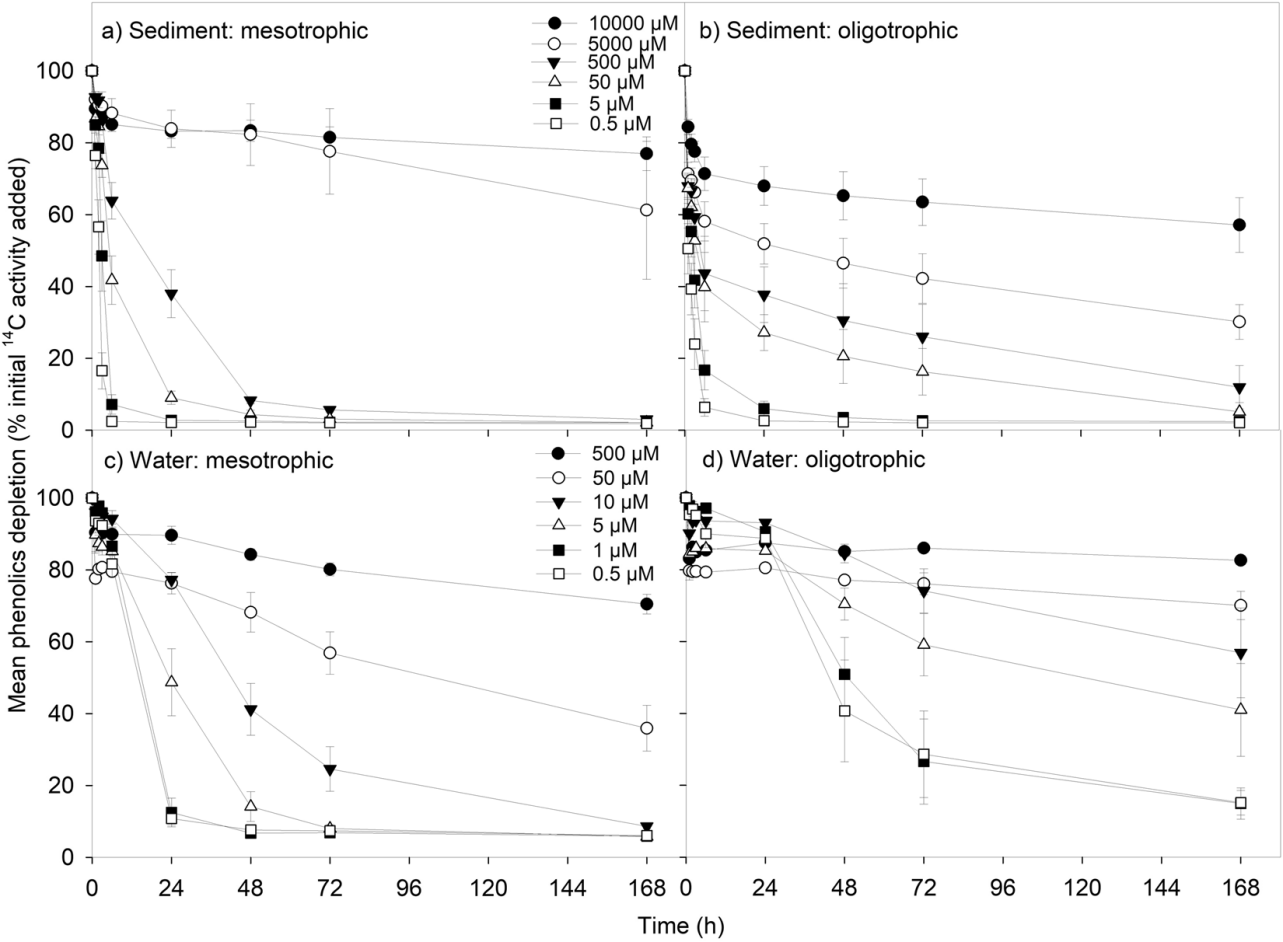
Effect of DOC concentration on the loss of ^14^C-labelled phenolic compounds for: (**a**) lowland improved grassland river sediments (mesotrophic), (**b**) upland peat bog sediments (oligotrophic), (**c**) lowland improved grassland river waters (mesotrophic), (**d**) upland improved grassland river waters (oligotrophic). Values represent means ± SEM (*n* = 3). Please note the legends are different for the top two panels (a and b) and bottom two panels (c and d) to represent the different concentration ranges found in each substrate type; sediment and water respectively. The legend is the same for the top two (**a** and **b**) and bottom two (**c** and **d**) panels respectively.

After incubation for 1, 2, 5, 24, 40, 48, 72 and 168 h, by which point ^14^C-compound depletion had plateaued, 0.5 mL subsamples were removed from the tubes, centrifuged to remove microbial cells (20,817 *g*, 3 min), and 0.25 mL of the supernatant placed into a scintillation vial. Destructive sampling was not possible due to the large number of samples (*n* = 6912), however samples were kept at stable conditions (in the dark, at 10 °C) and on a shaker to ensure they were well mixed over the course of the experiment, to prevent autochthonous C production and maintain diffusion within the samples. In addition, a headspace of at least 5 mL was maintained in order to prevent samples from becoming anaerobic. The results do not indicate that microbial growth took place as the depletion of ^14^C-labelled DOC compounds from solution did not follow a sigmoidal curve (Figs 1–4). The subsamples were then acidified with HCl (25 µL, 0.1 M), left to stand overnight and then vortexed to remove any remaining dissolved H^14^CO_3_/^14^CO_2_ present. The subsample was then mixed with Optiphase HiSafe scintillation cocktail (4 mL; PerkinElmer, Waltham, MA, USA) and the ^14^C quantified on a Wallac 1404 liquid scintillation counter with automated quench correction (Wallac EG&G, Milton Keynes, UK).

### Statistical analysis

Initial rates of uptake of the DOC groups were calculated as the percentage of added ^14^C depleted within 1 h. Lineweaver-Burke plots were used to estimate the Michaelis-Menten parameters V_max_, the maximum rate of DOC from solution and K_m_, the substrate concentration at which half the maximal uptake rate is achieved. Data analyses were conducted using SPSS 22.0 (IBM UK Ltd, Portsmouth, UK). Independent t-tests were used to determine any differences between sediment and water characteristics for each land cover type. Two-way mixed analysis of variance (ANOVA) was used to test for significant differences between sample type, land cover, DOC (sugars, amino acids organic acids, phenolic compounds) compound group and the concentration of the DOC compound group added. For comparisons of sediments and waters, only data from concentrations used in both water and sediment treatments were used in statistical analysis (Table S2). The significance level of the P-value was set at *p* ≤ 0.05. If the data did not meet the criteria of Mauchly’s test for sphericity, the Greenhouse-Geisser correction was applied to the P-value.

## Results

### Sediment and water characteristics

The water samples from the two contrasting stream types used in the study were found to differ more widely in their chemical properties than the sediment samples (Tables 1; S3). The pH, Electroconductivity (EC), total dissolved nitrogen, nitrate and orthophosphate values were significantly higher in samples from lowland mesotrophic sites, while DOC was found to be significantly higher in samples from the upland oligotrophic sites. These trends align with the peaty soils found in the upland oligotrophic soils and the manure and fertiliser nutrient-enriched soils in the lowland mesotrophic catchments (Emmett *et al*.^31^). For sediments, pH, EC and molybdate-reactive P values were higher in lowland mesotrophic sediments compared to to upland oligotrophic sediments, whilst moisture content, organic matter, total carbon and total nitrogen were higher for upland oligotrophic sediments. This is likely due to the higher levels of variation observed within this dataset.

**Table 1:** Chemical characteristics of the water and sediment samples used in the study.

	Lowland mesotrophic	Upland oligotrophic	F	*P*-value
**Water**				
pH	7.09 ± 0.08	4.20 ± 0.16	14	<0.001*
Electrical conductivity (μS cm^−1^)	191 ± 8	49 ± 5	13	<0.001*
Temperature (°C)	7.53 ± 0.37	9.00 ± 0.65	3	0.421
Dissolved organic C (mg C L^−1^)	2.86 ± 0.13	7.60 ± 0.62	6	<0.001*
Total free carbohydrates (mg C L^−1^)	0.11 ± 0.02	0.09 ± 0.02	1	0.45
Total phenols (mg C L^−1^)	2.27 ± 0.66	1.78 ± 0.74	0	0.667
Total dissolved N (mg N L^−1^)	2.13 ± 0.24	0.38 ± 0.02	7	<0.001*
NH_4_^+^ (mg N L^−1^)	0.05 ± 0.01	0.06 ± 0.01	0	0.818
NO_3_^−^ (mg N L^−1^)	1.73 ± 0.24	0.02 ± 0.00	7	<0.001*
Total free amino acids (mg N L^−1^)	0.10 ± 0.01	0.13 ± 0.01	2	0.142
Molybdate-reactive P (mg P L^−1^)	0.07 ± 0.01	0.03 ± 0.00	4	<0.001*
**Sediment**				
pH_(H2O)_	6.87 ± 0.06	4.75 ± 0.05	27	<0.001*
Electrical conductivity (μS cm^−1^)	37 ± 9	15 ± 2	4	0.002*
Moisture content (%)	40.0 ± 3.6	80.3 ± 3.6	8	<0.001*
Silt content (%)	27.6 ± 14.6	5.2 ± 1.3	2	0.263
Clay content (%)	10.2 ± 5.8	0.7 ± 0.3	2	0.243
Sand content (%)	62.3 ± 20.3	94.1 ± 1.6	2	0.257
Total C (mg C kg sediment^−1^)	7.19 ± 1.22	250 ± 41.7	6	<0.001*
Total free carbohydrates (mg C kg wet sediment^−1^)	0.59 ± 0.03	0.61 ± 0.08	0	0.829
Total phenols (mg C kg wet sediment^−1^)	4.05 ± 1.37	7.26 ± 2.58	1	0.358
Total N (mg N kg sediment^−1^)	1.14 ± 0.08	8.36 ± 1.28	6	<0.001*
NH_4_^+^ (mg N kg wet sediment^−1^)	11.7 ± 5.5	5.1 ± 1.8	1	0.272
NO_3_^−^ (mg N kg wet sediment^−1^)	0.26 ± 0.10	0.91 ± 0.26	2	0.061
Total amino acids (mg N kg wet sediment^−1^)	0.20 ± 0.02	0.20 ± 0.01	0	0.834
Molybdate-reactive P (mg P kg wet sediment^−1^)	2.05 ± 0.21	0.21 ± 0.05	9	<0.001*

Values represent means ± SEM, *n* = 9. *Denotes a significant *P*-value when comparing the two sites. The significance level was set at *P* < 0.05. All values for sediments are expressed on a dry weight basis unless otherwise stated.

Higher abundances of PLFAs were recovered from sediment samples in comparison to water samples (Table 2; Table S4). For waters, there were approximately half the amount of PLFAs of fungal origin in the upland oligotrophic sites in comparison to the lowland mesotrophic sites. No other taxa were found to differ. By contrast, approximately four times as many PLFAs were recovered from upland oligotrophic sediments in comparison to lowland mesotrophic sediments, which might reflect the higher abundance of submerged plants in the upland stream reaches. More PLFAs of gram positive bacterial origin were found in upland oligotrophic sediments than in their mesotrophic counterparts.

**Table 2:** Analysis of total mass of Phospholipid-derived fatty acids (PLFA) and taxonomic groups of concentrated water samples and freeze-dried sediment samples used in the study.

	Lowland eutrophic	Upland oligotrophic	F	*P*-value
**Water**				
Total PLFA biomass (nmol ml water^−1^)	0.07 ± 0.02	0.12 ± 0.07	1	0.475
Gram − bacteria (%)	51.2 ± 4.5	58.1 ± 4.3	1	0.329
Gram + bacteria (%)	33.0 ± 4.5	27.5 ± 3.9	1	0.399
Actinomycetes (%)	3.25 ± 1.37	2.23 ± 2.09	1	0.428
AM Fungi (%)	4.10 ± 4.71	5.50 ± 3.59	1	0.680
Fungi (%)	3.92 ± 0.26	1.86 ± 0.22	6	0.004*
Eukaryote (%)	4.48 ± 0.55	4.84 ± 1.43	0	0.824
**Sediment**				
Total PLFA biomass (nmol g sediment ^−1^)	152 ± 34	621 ± 180	3	0.048*
Gram − bacteria (%)	47.1 ± 2.1	47.8 ± 0.7	3	0.753
Gram + bacteria (%)	25.5 ± 0.8	30.1 ± 1.9	2	0.047*
Actinomycetes (%)	6.79 ± 1.02	8.27 ± 2.09	1	0.346
Fungi (%)	5.41 ± 2.78	4.51 ± 0.23	1	0.754
Eukaryote (%)	8.63 ± 1.73	6.35 ± 0.64	1	0.245

Values represent means ± SEM, *n* = 3. *Denotes a significant *P*-value when comparing the two sites. The significance level was set at *P* < 0.05.

### DOC uptake in sediment versus water

For all DOC compound groups, the highest maximal reaction rates (V_max_) were observed for mesotrophic sediments, which were three orders of magnitude higher in comparison to mesotrophic river waters (Fig. S3). No differences in V_max_ were observed between sediment and water from oligotrophic rivers. The K_m_ values for the different DOC groups were also lowest in sediments from mesotrophic rivers, indicating that a lower concentration of DOC is required to reach the maximum uptake rates.

Higher rates of initial rate of ^14^C-amino acid depletion (µmol cm^−3^ hour^−1^) were observed in sediments in comparison to waters (for comparable concentrations only) for both the oligotrophic and mesotrophic rivers (two-way ANOVA, *P* < 0.001). Whilst the mean initial rate of ^14^C-glucose depletion was also higher in sediments than in waters for comparable concentrations (<500 µM) for both mesotrophic and oligotrophic rivers (two-way ANOVA, *P* < 0.001), the amount of glucose remaining in sediment and water samples at the end of the experiment was not found to differ in oligotrophic rivers (two-way ANOVA, *P* = 0.873; Tables S5, S8).

For comparable concentrations of organic acids (<500 µM), the initial rate of ^14^C-labelled organic acid uptake was higher in both mesotrophic and oligotrophic sediments in comparison to waters from the same sites (two-way ANOVA, *P* < 0.001 for both). This corresponded with there being less organic acid remaining in solution for sediments in comparison to waters from mesotrophic sites (two-way ANOVA, *P* = 0.019; Tables S6,7).

In lowland mesotrophic sites there was no difference in the initial rate of phenolics depletion between sediments and waters (two-way ANOVA, *P* = 0.579), however, for upland oligotrophic sites the initial rate of phenolics depletion was higher in sediments in comparison to waters (two-way ANOVA, *P* < 0.001; Table S7). In contrast, for both oligotrophic and mesotrophic sites there were more phenolics remaining in solution at the end of the experiment in water samples compared to sediment samples where the same concentration was used (two-way ANOVA, *P* = 0.001 and *P* < 0.001 respectively).

### DOC uptake in two waters draining contrasting catchment types

#### ^14^C-labelled amino acid uptake

For sediments, the mean initial amino acid depletion rate was double in oligotrophic rivers in comparison to mesotrophic rivers (two-way ANOVA, *P* = 0.006; Fig. 1; Tables S6–8). However, oligotrophic sediments had double the amount of amino acids remaining at the end of the experiment compared to mesotrophic sediments (two-way ANOVA, *P* < 0.001; Tables S6–8). This result was driven by the two highest amino acid concentrations, where there was high initial amino acid depletion followed by a period of saturation.

There was no difference in the mean ^14^C-amino acid depletion rate detected for river waters, however, there was a significant interaction between the trophic state of the waters and amino acid concentration, driven by the difference in the amino acids remaining at the end of the assay at the highest concentration (500 µM) (two-way ANOVA, *P* = 0.715, *P* < 0.001 respectively). At the end of the experiment, there was double the amount of amino acids remaining in oligotrophic waters in comparison to mesotrophic waters (two-way ANOVA, *P* < 0.001; Tables S6–8).

#### ^14^C-labelled glucose uptake

Overall, two thirds of the initial ^14^C-glucose remained at the end of the experiment in samples from mesotrophic river sediments in comparison to oligotrophic river sediments (two-way ANOVA, *P* = 0.037; Fig. 2). The difference between the percentage of ^14^C-glucose remaining in waters from the two contrasting land cover types was greater, with ~23% more glucose remaining in solution for oligotrophic waters (two-way ANOVA, *P* = 0.020; Tables S6–8), despite the higher initial ^14^C-glucose depletion rate in sediments and waters from oligotrophic rivers (two-way ANOVA, *P* < 0.001).

#### ^14^C-labelled organic acids uptake

When the results for the two contrasting land cover types were compared, the initial organic acid depletion rate was ~60% higher in oligotrophic sediments than mesotrophic sediments, however, no difference was found between mesotrophic and oligotrophic waters (two-way ANOVA, *P* < 0.001 and *P* = 0.947 respectively; Fig. 3; Tables S6–8). There was also no difference in the amount of organic acids remaining in the mesotrophic and oligotrophic sediments by the end of the assay, whilst overall more organic acid compounds remained in the oligotrophic waters at the end of the experiment, in comparison to the mesotrophic waters (two-way ANOVA, *P* = 0.202 and *P* < 0.001 respectively; Fig. 3).

#### ^14^C-labelled phenolic compounds uptake

For upland oligotrophic sediments, despite an initial spike in mean phenolics uptake ~4 times higher than the initial rate observed in lowland mesotrophic sediments (two-way ANOVA, *P* < 0.001; Fig. 4), there was no effect of land cover at the end of the experiment (two-way ANOVA, *P* = 0.715). This can be attributed to the higher levels of variance observed in this dataset. In contrast, although there was no initial difference in phenolic compounds uptake rates between waters from the two land cover types (two-way ANOVA, *P* = 0.249), by the end of the experiment a greater uptake of phenolics had occurred in the lowland mesotrophic water in comparison to the upland oligotrophic waters (two-way ANOVA, *P* < 0.001).

### Uptake of DOC compound groups

The highest initial DOC uptake rate was observed for the phenolic compounds, for both mesotrophic sediments and oligotrophic waters (two-way ANOVA, *P* < 0.001). This was reflected in phenolics having the highest maximum velocity for the reaction for mesotrophic sediments (Fig. S4; Table S8). The mean initial ^14^C-labelled organic acid depletion rate in mesotrophic sediments was approximately one third of ^14^C-labelled glucose depletion rate, however, no difference was detected between organic acids and amino acids (two-way ANOVA, *P* < 0.001; Table S5). For oligotrophic sediments, the initial ^14^C-organic acid depletion rate was not different from that observed for amino acids and phenolic compounds. However, it was lower than the initial glucose depletion rate (two-way ANOVA, *P* < 0.001). The initial phenolic compounds depletion rate was higher than that of both amino acids and glucose (two-way ANOVA, *P* < 0.001). For both land cover types, the initial glucose uptake rate in sediments was half that of the amino acid uptake rate (two-way ANOVA, *P* < 0.001; Tables S5,6).

Although there was an overall significant effect of DOC compound group on the percentage DOC remaining at the end of the experiment, there was no difference between organic acids and glucose remaining by the end point (two-way ANOVA, *P* < 0.001 and *P* > 0.05 respectively; Table S6). However, there were less organic acids remaining at the end of the experiment compared to the phenolic compounds in the mesotrophic sediments. Despite the high initial depletion rates, glucose had the highest amount remaining by the end of the assay (two-way ANOVA, *P* < 0.001). The elevated phenolics uptake rates were also not sustained over the duration of the study; in both mesotrophic and oligotrophic sediments there were more phenolic compounds remaining in solution at the end point compared to glucose (two-way ANOVA, P < 0.001).

In contrast to the results for the sediment samples, the initial ^14^C-organic acid depletion rate in both mesotrophic and oligotrophic waters was not found to be different to the initial depletion rates of amino acids and glucose (two-way ANOVA, *P* < 0.001 for both). However, the initial ^14^C-glucose depletion rates in mesotrophic and oligotrophic waters were higher than the rates observed for ^14^C-labelled amino acids (two-way ANOVA, *P* < 0.001 in both cases; Fig. 2; Table S6). Although the initial phenolic compounds depletion rate was higher in upland oligotrophic waters, there was no difference in the initial phenolics depletion in lowland mesotrophic waters compared to the other compound groups (two-way ANOVA, *P* = 0.199). The combined mean percentage of both organic acids and glucose remaining at the end of the experiment was approximately half of the combined mean percentage of amino acids and phenolic compounds remaining in solution (two-way ANOVA, *P* < 0.001 for both).

## Discussion

The overall amino and organic acid processing rates were approximately double the rates for sediments in comparison to waters, for both mesotrophic and oligotrophic rivers, over the duration of the experiment. The elevated DOC processing rates in sediments highlight the importance of the hyporheic zone for in-stream carbon cycling; this includes sediments of the active channel and riparian zones, both of which provide a stable environment for higher microbial processing rates and comprise the main interface where surface and groundwaters mix^40^. As expected, there was a significant effect of ^14^C-compound concentration on DOC processing rates for the majority of treatments. The higher intrinsic nutrient loadings observed for sediments in comparison to waters are likely to be a contributing factor to the elevated processing rates; intrinsic DOC concentrations have previously been found to have a positive correlation with the in-stream organic matter processing rate^11,41,42^. Sediments were also found to have a higher microbial biomass compared to waters at both oligotrophic and mesotrophic sites in the current study, which may also increase their uptake capacity for DOC compounds. Alternatively, the greater depletion observed in these treatments could be ascribed to abiotic sorption of the compounds to the sediment’s solid phase, however this is known to be low, particularly for weakly or neutrally charged solutes and sediments with low cation exchange capacity such as those used here^43,44^. This was confirmed by control experiments performed as part of this study (Fig. S4).

In upland oligotrophic waters, glucose was initially processed more quickly in the water column than in the sediment at comparable concentrations, although a higher proportion of glucose added was processed in sediments over the whole experimental period. Phenolic compounds were also initially processed at higher rates in upland oligotrophic waters in comparison to upland oligotrophic sediments, although more phenolic compounds were processed in sediments overall, as observed for glucose. These results are in agreement with the earlier findings of Dawson and colleagues^45^ who reported in-stream processing of DOC in carbon-rich upland waters as a major factor governing DOC gradients on a spatial scale along the length of a lotic water body. ^14^C-glucose uptake has previously been used as a proxy for microbial activity^11,46^; therefore the higher initial rate of glucose processing in the oligotrophic water column could indicate that initially there is more microbial activity in oligotrophic waters in comparison to mesotrophic waters. It has previously been found that bacterial growth efficiency in terms of DOC utilisation can be higher in carbon-rich waters, compared to carbon-poor waters, which is in agreement with the above results^22^. However, the lag phase observed in sediments previously described could indicate that faster microbial growth is occurring in the sediments, allowing more rapid glucose processing.

For both sediment and water, our study showed that microbial communities from lowland mesotrophic rivers were able to process higher DOC concentrations than those from upland oligotrophic rivers, with the exception of organic acids and phenolic compounds in sediments only. We hypothesise that this could be attributed to a range of factors including: (i) the higher background inorganic nutrient concentrations in lowland mesotrophic waters, thereby removing metabolic constraints on substrate uptake and microbial growth^28,47^; (ii) the elevated high MW humic substances concentrations in the oligotrophic waters which may limit biological activity via binding and inhibiting free enzymes responsible for substrate catalysis^5,30,48^; (iii) the binding of the added substrates to DOC in the water, removing these from solution early in the experiment and thus preventing microbial uptake, (iv) the binding of intrinsic DOC to the organisms present, thereby suppressing membrane bound transport systems^49,50^; or (v) the higher concentration of organisms in the mesotrophic waters and sediments in comparison to those from the oligotrophic environment, as shown by the PLFA data.

Initially, there was a slightly higher rate of DOC uptake in oligotrophic waters, however, this was not sustained over the duration of the experiment. This, alongside the higher inorganic nutrient concentrations typically present in the mesotrophic waters, lends support to hypothesis (i), but does not support hypothesis (v) based on our PLFA data. In contrast, based on the abundant microbial transport systems for LMW substrates discovered by metagenomic sequencing (which suggests largely intracellular LMW DOC breakdown) we do not favour hypothesis (ii). Similarly, we discount (iii) as most of our C substrates have neutral charge at the pH values used here and are therefore unlikely to interact strongly with intrinsic DOC present in the samples or with mineral surfaces. Hypothesis (iv) cannot be critically evaluated in our study and further work would need to be undertaken to evaluate its significance.

A lag phase in substrate uptake was observed for mesotrophic rivers, such that DOC processing was initially quicker in oligotrophic rivers; this was particularly evident in sediments. This lag phase in mesotrophic rivers could be attributed to microbial growth or the microbial community becoming more active over time (e.g. resuscitation from a starvation/viable-but-non-culturable state)^51^. Previous studies have found that mixing sediment with water containing a lower concentration of DOC than in the natural overlying waters can halt growth or reduce the biomass by approximately 50% over the short term, with the community reaching a new steady state after 4 days^52^. In the current study, this lag phase generally did not exceed 48 hours and was followed by an increase in the DOC uptake rate.

The fastest initial DOC processing rate observed was for amino acids in oligotrophic sediments at 0.23 ± 0.09 µmol cm^−3^ h^−1^. A higher proportion of amino acids were processed in sediments than in waters from both land cover types. Previously we have ascribed the rapid uptake of amino acids as relating to its labile DOC (i.e. readily used by a C-limited microbial community)^23^, but it is also a source of DON. ^14^C-labelled amino acid studies have found that the C skeleton produced during intracellular amino acid processing (e.g. pyruvate) can be excreted with intracellular N increasing following amino acid uptake^53^. This is supported by the percentage of amino acids remaining in solution never falling below 20% for waters in particular; a trend which has also been observed in previous studies^23,54^. Catchment-scale studies of DOM processing have found that amino acid uptake capacity was the highest in peat-influenced streams, which is likely linked to the N limitation characteristic of these ecosystems^55^. However, the greatest overall DOC loss from solution was observed for organic acids and glucose in mesotrophic sediments. All of the initial DOC uptake rates measured were within the same order of magnitude as those found for ^14^C-labelled glucose uptake in soils and for glucose^26^ and acetate^56^ by bacteria and algae in aquatic environments.

A study of ^14^C-glucose rates versus ^14^C-phenol uptake in humic and clear waters, which also measured bacterial abundance alongside the assays, found that glucose uptake peaked during the exponential phase of bacterial growth, with the biggest peak seen in clear waters^22^. Although approximately the same amount of glucose was processed over the duration of the experiment, higher bacterial growth efficiency was observed in humic waters, with the bacterial biomass reaching a higher abundance relative to clear water and mixed clear water/humic water samples. Microbes from humic waters began to process phenol once the glucose had been almost completely utilised, indicating that they will preferentially use more labile DOC, but can also adapt to use more aromatic compounds. The usage of phenol by microbes from clear waters did not exceed the limit of detection for the duration of the experiment^22^. Our results appear to mirror this earlier study, with higher sustained glucose uptake rates in comparison to phenolic compounds over the course of the experiment, and an initial lag-phase evident before glucose uptake begins, indicating that this too could coincide with a period of microbial growth. However, more evidence of phenolic compound processing was observed in this study, which may relate to the fact that our samples were collected at slightly warmer temperatures than in the work of Tranvik and Höfle^22^, or that the phenolic compounds used in this study contained more aliphatic bonds than phenol, which were used in the earlier study.

The phenolic compound processing in the current study may be limited by abiotic factors such as photodegradation, as samples were incubated in the dark. Previous studies have shown that photodegradation alone, in the absence of microbial processing, can result in the production of labile DOC from larger, humic-like compounds, which are more bioavailable to the microbial community^57^. Photochemical degradation of DOC compounds is particularly important in upland mountain, heath and bog habitats where the amount of shading by riparian vegetation is lowest; this can also act to influence water temperature, moderating biotic processes^58^.

We conclude that the higher inorganic nutrient concentrations and greater microbial biomass of sediments allows more rapid processing of LMW DOC compounds, particularly at higher background DOC enrichment. For mesotrophic sediments in particular, the greater availability of N and P to the stream biota may remove the inorganic nutrient limitation barrier on DOC uptake, providing them with a greater capacity for in-stream DOC processing. In comparison, oligotrophic rivers processed less DOC than the mesotrophic rivers; initial DOC processing primarily took place in the water column in oligotrophic rivers, although the sediments processed more DOC overall, with a preference for the simplest compounds (amino acids, glucose). If DOC is not processed fully in the uplands and DOC export from peatlands continues to increase over the coming years, this may exacerbate problems in downstream lowland areas, which has implications for future water quality management.

## Supplementary information

Supplementary Information

## Data Availability

The datasets generated during and/or analysed during the current study are available from the corresponding author on reasonable request.
