# Supplementary material for: Microbial uptake kinetics of dissolved organic carbon (DOC) compound groups from river water and sediments
Source: Sci Rep. 2019 Aug 2;9:11229. doi: 10.1038/s41598-019-47749-6 (PMC6677892; doi:10.1038/s41598-019-47749-6)
Supplement: Supplementary file 1 — Supplementary Information [file 41598_2019_47749_MOESM1_ESM.pdf]

Supplementary Information

Microbial uptake kinetics of dissolved organic carbon (DOC) compound groups from river water and sediments

Francesca L. Brailsford, Helen C. Glanville, Peter N. Golyshin , Penny J. Johnes, Christopher A. Yates and Davey L. Jones

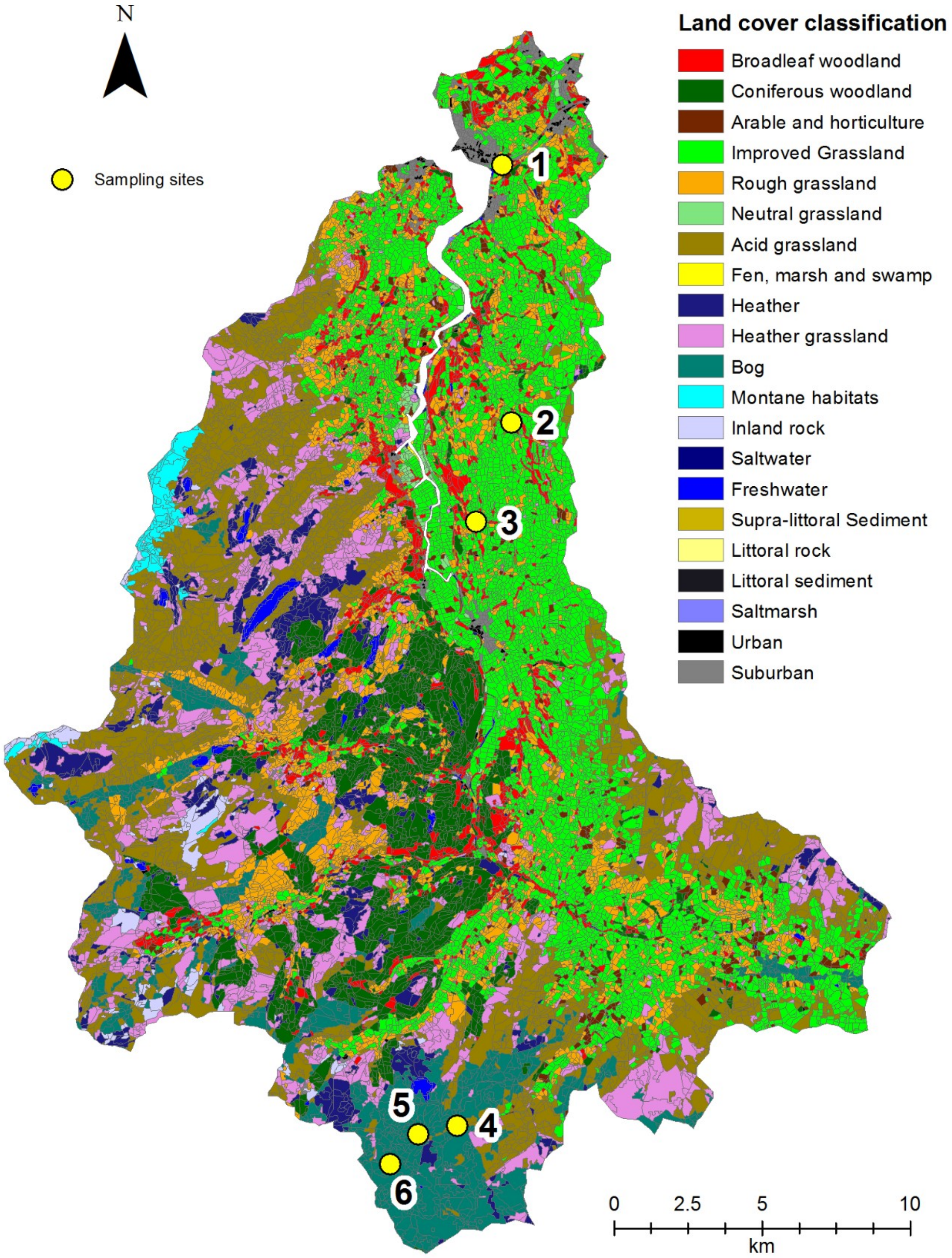

Fig. S1 Land cover map of the Conwy catchment with lowland improved grassland sites (1-3) and upland peat bog sites (4-6) indicated. Created with ArcGIS Hydrology toolbox (ESRI 2018. Version 10 Redlands, CA) using LCM2007 data provided by the Centre for Ecology and Hydrology (Emmett et al. 2016).

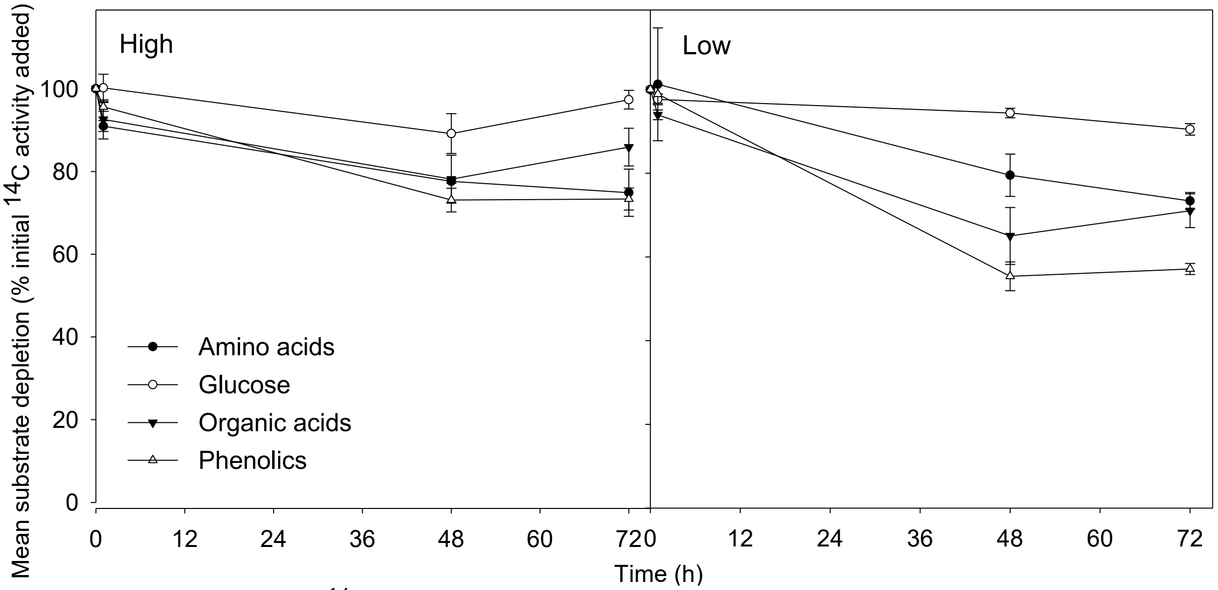

Fig. S2 Abiotic loss of ^{14}C-labelled amino acids, glucose, organic acids and phenolics compounds from river sediments sterilised with formaldehyde. The highest and lowest substrate concentrations were used (5000 µm and 0.1 µm for ^{14}C-labelled glucose and 10000 µm and 0.5 µm for ^{14}C-labelled amino acids, organic acids and phenolics. Values represent means ± SEM (*n* = 3).

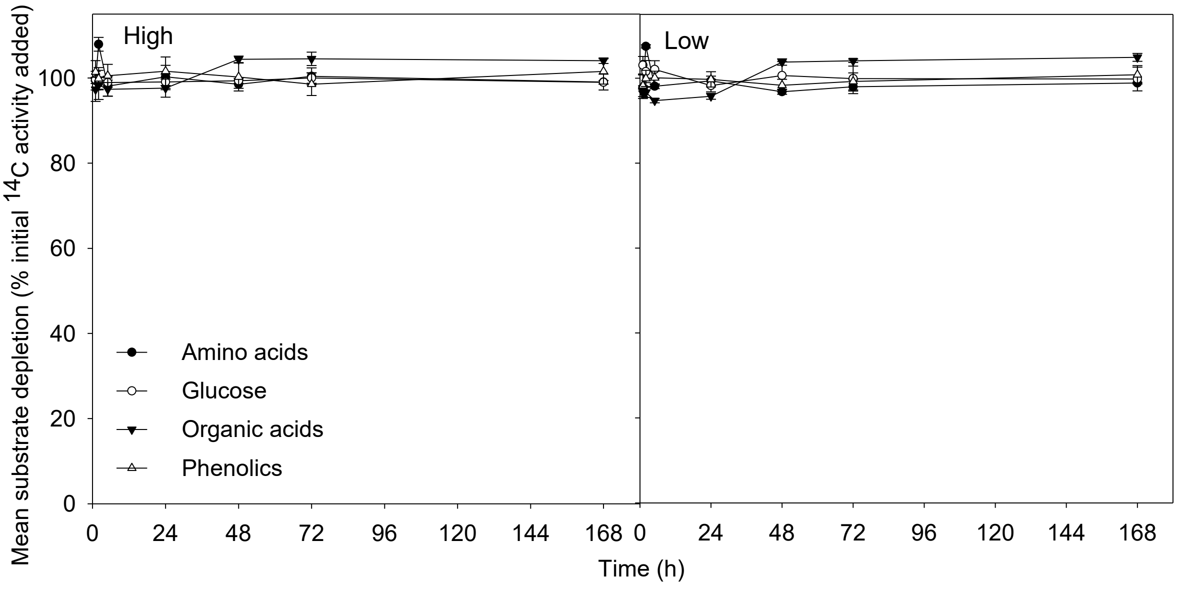

Fig. S3 Abiotic loss of ^{14}C-labelled amino acids, glucose, organic acids and phenolics compounds from water. The highest and lowest substrate concentrations were used (50 µm and 0.1 µm for ^{14}C-labelled glucose and 500 µm and 0.5 µm for ^{14}C-labelled amino acids, organic acids and phenolics. Values represent means ± SEM (*n* = 3).

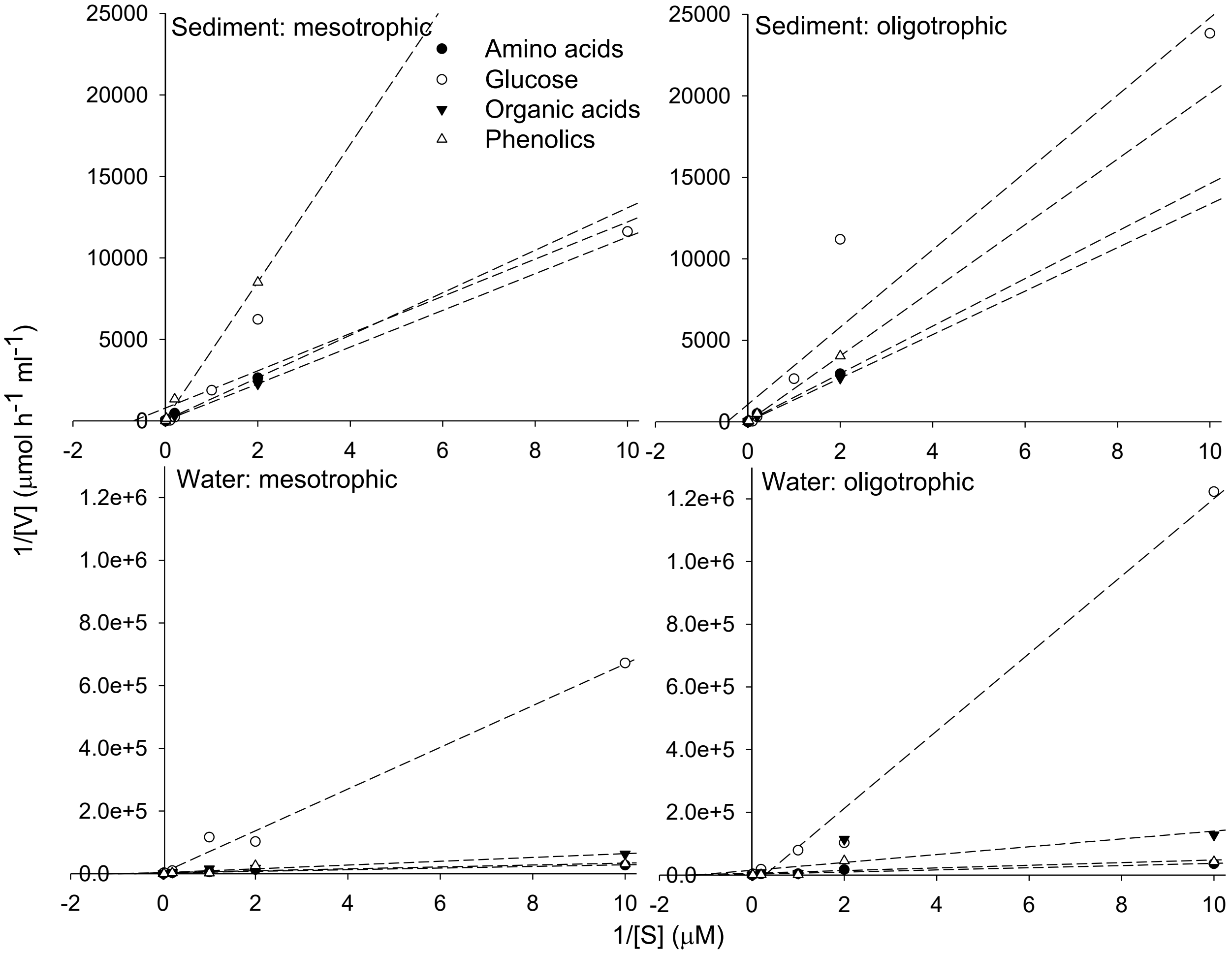

Fig. S4 Lineweaver-Burke plots for amino acids, glucose, organic acids and phenolics compounds for: lowland improved grassland river sediments (mesotrophic), upland peat bog sediments (oligotrophic), lowland improved grassland river waters (mesotrophic) and upland improved grassland river waters (oligotrophic).

Table S1 Compounds used in the kinetics experiments.

Functional group	Compound	Isotope	Supplier	Lot Number
**Sugars**	Glucose	^{14}C-[U]-glucose	Perkin Elmer	3632475
	Alanine	^{14}C-[U]-amino acids	Perkin Elmer	2132273
	Arginine	^{14}C-[U]-amino acids	Perkin Elmer	2132273
	Aspartate	^{14}C-[U]-amino acids	Perkin Elmer	2132273
	Glutamate	^{14}C-[U]-amino acids	Perkin Elmer	2132273
	Glycine	^{14}C-[U]-amino acids	Perkin Elmer	2132273
	Isoleucine	^{14}C-[U]-amino acids	Perkin Elmer	2132273
**Amino acids**	Lysine	^{14}C-[U]-amino acids	Perkin Elmer	2132273
	Methionine	^{14}C-[U]-amino acids	Perkin Elmer	2132273
	Phenylalanine	^{14}C-[U]-amino acids	Perkin Elmer	2132273
	Proline	^{14}C-[U]-amino acids	Perkin Elmer	2132273
	Serine	^{14}C-[U]-amino acids	Perkin Elmer	2132273
	Tyrosine	^{14}C-[U]-amino acids	Perkin Elmer	2132273
	Valine	^{14}C-[U]-amino acids	Perkin Elmer	2132273
	Acetic acid	^{14}C-[U]-acetic acid	Perkin Elmer	1931680
**Organic acids**	Citric acid	^{14}C-[U]-citric acid	Perkin Elmer	3604237
	Malic acid	^{14}C-[U]-malic acid	American Radiolabeled Chemicals (ARC)	150508
	P-coumaric acid	^{14}C-[U]-*P*-coumaric acid	American Radiolabeled Chemicals (ARC)	161117
**Phenolics**	Salicylic acid	^{14}C-[U]-salicylic acid	American Radiolabeled Chemicals (ARC)	070502
	Vanillic acid	^{14}C-[U]-vanillic acid	American Radiolabeled Chemicals (ARC)	160311

Table S2 Concentrations of functional groups used in the kinetic experiments.

	Sediments				Waters			
	**Amino acids**	**Sugars**	**Organic acids**	**Phenolics**	**Amino acids**	**Sugars**	**Organic acids**	**Phenolics**
**Concentration (μM)**	10000	-	10000	10000	500	-	500	500
	5000	5000	5000	5000	50	50	50	50
	500	500	500	500	-	10	-	-
	50	50	50	50	5	5	5	5
	5	5	5	5	1	1	1	1
	0.5	0.5	0.5	0.5	0.5	0.5	0.5	0.5
	-	0.1	-	-	0.1	0.1	0.1	0.1

Table S3 Mean chemical characteristics of the water samples used in the study. Values represent mean ± SEM (n = 3) except for soil texture analysis. BLD, below limit of detection.

	Lowland mesotrophic sites			Upland oligotrophic sites		
	**1**	**2**	**3**	**4**	**5**	**6**
**Water**						
$pH_{(H2O)}$	7.27 ± 0.20	6.87 ± 0.05	7.15 ± 0.10	4.34 ± 0.35	4.14 ± 0.37	4.11 ± 0.33
Electrical conductivity (µS cm^{-1})	201 ± 6	207 ± 12	163 ± 15	46 ± 6	51 ± 15	49 ± 9
Dissolved organic C (mg C L^{-1})	2.88 ± 0.56	2.58 ± 0.35	3.11 ± 0.46	7.48 ± 1.31	6.43 ± 1.33	8.89 ± 1.53
Total free carbohydrates (mg C L^{-1})	0.08 ± 0.03	0.10 ± 0.03	0.16 ± 0.04	0.08 ± 0.04	0.12 ± 0.05	0.06 ± 0.01
Total phenols (mg C L^{-1})	0.49 ± 0.09	1.65 ± 0.00	3.67 ± 0.26	3.24 ± 0.00	1.06 ± 0.18	BLD
Total dissolved N (mg N L^{-1})	1.33 ± 0.08	2.78 ± 0.18	2.29 ± 0.25	0.36 ± 0.01	0.38 ± 0.05	0.41 ± 0.02
NH_4^+ (mg N L^{-1})	0.05 ± 0.02	0.05 ± 0.02	0.06 ± 0.02	0.06 ± 0.02	0.06 ± 0.02	0.05 ± 0.02
NO_3^- (mg N L^{-1})	0.96 ± 0.16	2.31 ± 0.23	1.91 ± 0.37	0.01 ± 0.01	0.02 ± 0.01	0.02 ± 0.02
Total free amino acids (mg N L^{-1})	0.10 ± 0.04	0.11 ± 0.02	0.10 ± 0.01	0.10 ± 0.01	0.14 ± 0.03	0.14 ± 0.01
Molybdate-reactive P (mg P L^{-1})	0.07 ± 0.01	0.09 ± 0.01	0.05 ± 0.01	0.02 ± 0.01	0.03 ± 0.00	0.04 ± 0.00
**Sediment**						
$pH_{(H2O)}$	6.90 ± 0.06	6.85 ± 1.10	6.84 ± 0.17	4.69 ± 10.0	4.87 ± 0.14	4.70 ± 2.04
Electrical conductivity (µS cm^{-1})	55 ± 4	29 ± 2	27 ± 4	15 ± 3	18 ± 5	10 ± 0
Moisture content (%)	44.4 ± 9.2	40.3 ± 6.4	35.2 ± 2.3	80.9 ± 6.2	76.1 ± 9.4	83.8 ± 2.1
Silt content (%)	56.53	15.85	10.33	3.32	4.51	7.69
Clay content (%)	21.7	4.31	4.44	0.2	0.55	1.34
Sand content (%)	21.77	79.84	85.23	96.48	94.94	90.97
Total C (mg C kg dry $sediment^{-1}$)	8.09 ± 0.70	3.11 ± 0.33	10.4 ± 1.88	414 ± 12.5	144 ± 7.2	194 ± 2.9
Total free carbohydrates (mg C wet $sediment^{-1}$)	0.56 ± 0.05	0.72 ± 0.03	0.53 ± 0.04	0.65 ± 0.03	0.42 ± 0.04	0.76 ± 0.23
Total phenols (mg C kg wet $sediment^{-1}$)	3.08 ± 2.88	11.4 ± 5.7	4.97 ± 2.96	5.91 ± 2.28	2.86 ± 0.00	3.30 ± 0.85
Total N (mg N kg dry $sediment^{-1}$)	1.12 ± 0.15	0.95 ± 0.08	1.36 ± 0.06	13.4 ± 0.17	5.15 ± 0.32	6.53 ± 0.15
NH_4^+ (mg N kg wet $sediment^{-1}$)	2.55 ± 0.88	1.19 ± 0.01	1.38 ± 0.06	0.99 ± 0.10	1.60 ± 0.37	1.84 ± 0.89
NO_3^- (mg N kg wet $sediment^{-1}$)	0.41 ± 0.28	0.11 ± 0.04	0.20 ± 0.04	1.70 ± 0.00	0.81 ± 0.00	0.19 ± 0.00
Total free amino acids (mg N kg wet $sediment^{-1}$)	0.27 ± 0.03	0.13 ± 0.02	0.19 ± 0.01	0.18 ± 0.01	0.21 ± 0.03	0.22 ± 0.02
Molybdate-reactive P (mg P kg wet $sediment^{-1}$)	2.59 ± 0.26	1.64 ± 0.43	1.92 ± 0.19	0.20 ± 0.04	0.12 ± 0.04	0.30 ± 0.13

Table S4 Analysis of total mass of phospholipid-derived fatty acids (PLFA) and taxonomic groups of concentrated water samples and freeze-dried sediment samples used in the study. Sediment values represent mean ± SEM (*n* = 3).

	**Lowland mesotrophic sites**			**Upland oligotrophic sites**		
	**1**	**2**	**3**	**4**	**5**	**6**
**Water**						
Total PLFA biomass (nmol ml water^{-1})	0.04	0.08	0.08	0.04	0.25	0.07
Gram – bacteria (%)	50	44.1	59.5	55	66.7	52.7
Gram + bacteria (%)	30.7	41.7	26.7	28.8	20.1	33.4
Actinomycetes (%)	3.97	3.08	2.71	4.42	1.13	1.16
Fungi (%)	4.22	4.14	3.4	1.43	2.1	2.05
Eukaryote (%)	5.54	3.7	4.19	3.09	7.68	3.75
**Sediment**						
Total PLFA biomass (nmol g sediment^{-1})	117 ± 54	239 ± 55	100 ± 1	1134 ± 186	531 ± 38	199 ± 47
Gram - bacteria (%)	43.8 ± 6.1	46.7 ± 1.6	50.7 ± 0.3	47.2 ± 1.8	48.4 ± 0.5	47.8 ± 1.6
Gram + bacteria (%)	24.5 ± 1.6	24.8 ± 1.7	27.2 ± 0.4	28.3 ± 2.7	32.9 ± 5.3	29.0 ± 1.9
Actinomycetes (%)	5.25 ± 1.64	5.59 ± 1.14	9.53 ± 0.54	8.79 ± 0.85	6.45 ± 3.18	9.57 ± 1.32
Fungi (%)	11.0 ± 8.1	3.6 ± 1.2	1.7 ± 0.0	4.5 ± 0.3	4.2 ± 0.5	4.9 ± 0.4
Eukaryote (%)	10.2 ± 1.0	11.3 ± 3.9	4.4 ± 1.1	7.6 ± 0.5	5.6 ± 1.8	5.8 ± 0.6

Table S5 Results from a two-way ANOVA for each isotopically-labelled nutrient, land-cover and sample type for assay end points (% of initial activity remaining). * Denotes a significant *P*-value. The significance level was set at $P < 0.05$.

Sample type	Nutrient	Effect of land-cover		Effect of concentration		Interaction concentration × land-cover	
		F	*P*-value	F	*P*-value	F	*P*-value
Sediment	^{14}C amino acids	28	<0.001*	58	<0.001*	14	<0.001*
Sediment	^{14}C glucose	16	0.001*	55	<0.001*	3	0.032*
Sediment	^{14}C organic acids	2	0.202	58	<0.001*	11	<0.001*
Sediment	^{14}C phenolics	18	<0.001*	14	<0.001*	1	0.477
Water	^{14}C amino acids	95	<0.001*	61	<0.001*	10	<0.001*
Water	^{14}C glucose	14	<0.001*	14	<0.001*	14	<0.001*
Water	^{14}C organic acids	482	<0.001*	195	<0.001*	114	<0.001*
Water	^{14}C phenolics	52	<0.001*	39	<0.001*	4	0.009*

Sample type	Land-cover	Effect of functional group		Effect of concentration		Interaction concentration × functional group	
		F	*P*-value	F	*P*-value	F	*P*-value
Sediment	Lowland mesotrophic	92	<0.001*	49	<0.001*	3	<0.001
Sediment	Upland oligotrophic	4	0.018*	42	<0.001*	5	<0.001*
Water	Lowland mesotrophic	70	<0.001*	135	<0.001*	20	<0.001*
Water	Upland oligotrophic	10	<0.001*	81	<0.001*	2	0.028*

Land-cover	Nutrient	Effect of sample type		Effect of concentration		Interaction concentration × sample type	
		F	*P*-value	F	*P*-value	F	*P*-value
Lowland mesotrophic	^{14}C amino acids	1	0.424	23	<0.001*	30	<0.001*
Lowland mesotrophic	^{14}C glucose	7	0.019*	9	0.001*	13	<0.001*
Lowland mesotrophic	^{14}C organic acids	580	<0.001*	8	0.002*	7	0.003*
Lowland mesotrophic	^{14}C phenolics	99	<0.001*	80	<0.001*	75	<0.001*
Upland oligotrophic	^{14}C amino acids	314	<0.001*	180	<0.001*	188	<0.001*
Upland oligotrophic	^{14}C glucose	0	0.873	48	<0.001*	1	0.524
Upland oligotrophic	^{14}C organic acids	1	0.348	150	<0.001*	127	<0.001*
Upland oligotrophic	^{14}C phenolics	17	0.001*	18	<0.001*	13	<0.001*

Table S6 Results from a two-way ANOVA for each isotopically-labelled nutrient, land-cover and sample type for initial rate of activity (µmol cm^{-3} h^{-1}).* Denotes a significant *P*-value. The significance level was set at $P < 0.05$.

Sample type	Nutrient	Effect of land-cover		Effect of concentration		Interaction concentration × land-cover	
		F	*P*-value	F	*P*-value	F	*P*-value
Sediment	^{14}C amino acids	9	0.006*	33	<0.001*	5	0.003*
Sediment	^{14}C glucose	5	0.037*	123	<0.001*	5	0.003*
Sediment	^{14}C organic acids	18	<0.001*	105	<0.001*	6	0.010*
Sediment	^{14}C phenolics	31	<0.001*	89	<0.001*	12	<0.001*
Water	^{14}C amino acids	0.136	0.715	5	0.002*	0	0.991
Water	^{14}C glucose	6	0.020*	1011	<0.001*	9	<0.001*
Water	^{14}C organic acids	0	0.947	77	<0.001*	0	1.000
Water	^{14}C phenolics	2	0.189	23	<0.001*	2	0.139
**Sample type**	**Land-cover**	**Effect of functional group**		**Effect of concentration**		**Interaction concentration × functional group**	
		F	*P*-value	F	*P*-value	F	*P*-value
Sediment	Lowland mesotrophic	9	<0.001*	113	<0.001*	5	<0.001*
Sediment	Upland oligotrophic	20	<0.001*	142	<0.001*	9	<0.001*
Water	Lowland mesotrophic	2	0.199	13	<0.001*	1	0.429

Water	Upland oligotrophic	60	<0.001*	238	<0.001*	48	<0.001*

Land-cover	Nutrient	Effect of sample type		Effect of concentration		Interaction concentration × sample type	
		F	*P*-value	F	*P*-value	F	*P*-value
Lowland mesotrophic	^{14}C amino acids	5	0.036*	9	<0.001*	2	0.226
Lowland mesotrophic	^{14}C glucose	43	<0.001*	40	<0.001*	29	<0.001*
Lowland mesotrophic	^{14}C organic acids	61	<0.001*	174	<0.001*	29	<0.001*
Lowland mesotrophic	^{14}C phenolics	0	0.579	8	0.001*	0	0.923
Upland oligotrophic	^{14}C amino acids	21	<0.001	22	<0.001*	12	<0.001*
Upland oligotrophic	^{14}C glucose	136	<0.001*	148	<0.001*	101	<0.001*
Upland oligotrophic	^{14}C organic acids	67	<0.001*	117	<0.001*	38	<0.001*
Upland oligotrophic	^{14}C phenolics	26	<0.001*	209	<0.001*	20	<0.001*

Table S7 Results from a two-way ANOVA for each isotopically-labelled nutrient, land-cover and sample type for initial rate of activity (μmol cm^{-3} h^{-1}).* Denotes a significant *P*-value. The significance level was set at $P < 0.05$.

Land-cover	Sample type	Concentration (μM)	End point (%) Amino acids	Glucose	Organic acids	Phenolics
Lowland mesotrophic	Sediment	10000	47.1 ± 0.1	-	76.1 ± 2.7	81.4 ± 4.7
		5000	31.2 ± 1.3	72.8 ± 6.2	51.3 ± 4.1	73.1 ± 11.8
		500	25.5 ± 0.3	46.1 ± 12.2	33.7 ± 0.2	47.6 ± 0.3
		50	27.5 ± 0.3	7.4 ± 0.1	33.0 ± 0.1	46.7 ± 0.4
		5	27.8 ± 0.3	8.1 ± 0.0	33.1 ± 0.1	46.8 ± 0.3
		0.5	28.3 ± 0.3	8.3 ± 0.0	33.3 ± 0.0	46.5 ± 0.1
		0.1	-	8.4 ± 0.1	-	-
Upland oligotrophic	Sediment	10000	66.3 ± 3.3	-	72.2 ± 7.8	71.0 ± 9.2
		5000	57.8 ± 7.4	90.7 ± 2.4	54.8 ± 6.9	44.0 ± 9.1
		500	25.8 ± 0.4	86.2 ± 1.8	38.5 ± 4.6	38.7 ± 6.0
		50	27.0 ± 0.4	28.8 ± 16.4	34.3 ± 0.9	37.1 ± 2.6
		5	26.7 ± 0.2	11.7 ± 1.8	35.0 ± 1.3	34.3 ± 0.1
		0.5	27.1 ± 0.2	10.4 ± 0.6	34.7 ± 1.0	34.0 ± 0.2
		0.1	-	9.9 ± 0.1	-	-
Lowland mesotrophic	Water	500	50.0 ± 4.2	-	16.4 ± 4.2	70.5 ± 2.8
		50	25.5 ± 5.1	6.3 ± 0.7	5.0 ± 0.4	35.9 ± 6.4
		10	13.8 ± 0.5	6.0 ± 0.6	4.9 ± 0.5	8.6 ± 0.7
		5	14.0 ± 0.5	5.7 ± 0.7	4.7 ± 0.3	5.8 ± 0.3
		1	13.1 ± 0.5	5.7 ± 0.6	4.8 ± 0.4	5.9 ± 0.2
		0.5	14.2 ± 0.2	5.2 ± 0.4	4.4 ± 0.2	6.1 ± 0.2
		0.1	-	6.8 ± 0.6	-	-

Upland oligotrophic	Water	500	75.2 ± 1.0	-	81.2 ± 2.9	82.6 ± 0.9
		50	68.2 ± 4.0	79.0 ± 4.5	50.2 ± 1.3	70.0 ± 4.0
		10	44.5 ± 9.0	38.0 ± 14.6	15.6 ± 2.4	56.8 ± 12.5
		5	20.3 ± 1.2	21.3 ± 8.3	9.0 ± 1.2	41.0 ± 13.0
		1	19.3 ± 1.2	9.9 ± 0.8	9.0 ± 0.5	14.9 ± 4.4
		0.5	20.7 ± 0.8	12.9 ± 0.6	7.9 ± 0.7	15.1 ± 3.4
		0.1	-	13.0 ± 0.2	-	-

**Land-cover**	**Sample type**	**Concentration (µM)**	**Initial rate (µmol cm^{-3} h^{-1})**			
			**Amino acids**	**Glucose**	**Organic acids**	**Phenolics**
Lowland mesotrophic	Sediment	10000	0.45815 ± 0.13368	-	0.63589 ± 0.06816	1.05436 ± 0.11036
		5000	0.12552 ± 0.04502	0.27064 ± 0.03011	0.33935 ± 0.09734	0.39688 ± 0.07032
		500	0.03335 ± 0.01149	0.03152 ± 0.00472	0.07488 ± 0.00685	0.03657 ± 0.00786
		50	0.01277 ± 0.00165	0.00438 ± 0.00201	0.01275 ± 0.00035	0.00664 ± 0.00025
		5	0.00214 ± 0.00014	0.00053 ± 0.00018	0.00316 ± 0.00020	0.00075 ± 0.00012
		0.5	0.00038 ± 0.00003	0.00016 ± 0.00005	0.00044 ± 0.00002	0.00012 ± 0.00002
		0.1	-	0.00009 ± 0.00001	-	-
Upland oligotrophic	Sediment	10000	1.02168 ± 0.16661	-	0.98712 ± 0.09294	1.55920 ± 0.21682
		5000	0.26823 ± 0.11391	0.40233 ± 0.05102	0.63706 ± 0.05637	1.43057 ± 0.15824
		500	0.06761 ± 0.01459	0.03273 ± 0.00283	0.11430 ± 0.01074	0.16049 ± 0.01572
		50	0.01513 ± 0.00182	0.00324 ± 0.00026	0.01873 ± 0.00325	0.01634 ± 0.00210
		5	0.00198 ± 0.00030	0.00038 ± 0.00019	0.00308 ± 0.00054	0.00199 ± 0.00020
		0.5	0.00034 ± 0.00004	0.00009 ± 0.00002	0.00037 ± 0.00006	0.00025 ± 0.00003
		0.1	-	0.00004 ± 0.00002	-	-
Lowland mesotrophic	Water	500	0.01340 ± 0.00816	-	0.03042 ± 0.00323	0.04803 ± 0.02653
		50	0.00243 ± 0.00039	0.00260 ± 0.00014	0.00138 ± 0.00044	0.01118 ± 0.00076
		10	0.00034 ± 0.00008	0.00032 ± 0.00003	0.00021 ± 0.00008	0.00039 ± 0.00024
		5	0.00019 ± 0.00005	0.00010 ± 0.00002	0.00006 ± 0.00006	0.00051 ± 0.00026
		1	0.00008 ± 0.00001	0.00001 ± 0.00001	0.00002 ± 0.00001	0.00004 ± 0.00001
		0.5	0.00004 ± 0.00001	0.00001 ± 0.00000	0.00006 ± 0.00002	0.00003 ± 0.00002

		0.1	-	0.00000 ± 0.00000	-	-
Upland oligotrophic	Water	500	0.01022 ± 0.00541	-	0.03057 ± 0.00603	0.08454 ± 0.00370
		50	0.00198 ± 0.00025	0.00310 ± 0.00009	0.00171 ± 0.00018	0.01014 ± 0.00132
		10	0.00031 ± 0.00009	0.00030 ± 0.00002	0.00013 ± 0.00004	0.00099 ± 0.00008
		5	0.00026 ± 0.00007	0.00006 ± 0.00001	0.00017 ± 0.00011	0.00078 ± 0.00005
		1	0.00006 ± 0.00000	0.00001 ± 0.00001	0.00001 ± 0.00001	0.00002 ± 0.00002
		0.5	0.00003 ± 0.00001	0.00001 ± 0.00000	0.00001 ± 0.00001	0.00002 ± 0.00000
		0.1	-	0.00000 ± 0.00000	-	-

Table S8 Parameters derived from Lineweaver-Burke plots based on kinetics data for ^{14}C amino acids, glucose, organic acids and phenolics. V_{max} is the maximum reaction velocity (µmol h^{-1} mL^{-1}) and K_m (Michaelis-Menten constant) is the substrate concentration at which half Vmax can be achieved (µM).

	Sediment mesotrophic			Sediment oligotrophic			Water mesotrophic			Water oligotrophic		
	V_{max}	K_m	r^2	V_{max}	K_m	r^2	V_{max}	K_m	r^2	V_{max}	K_m	r^2
Amino acids	23.2	0.02	0.99	0.0004	1.03	0.92	0.0199	28.99	1.00	0.0004	1.41	0.93
Glucose	1.43	0.00	0.89	0.0002	16.6	0.99	0.0009	2.216	0.92	0.0000	3.59	0.99
Organic acids	36.1	0.03	1.00	0.0003	1.62	0.98	0.0564	75.34	1.00	0.0001	0.83	0.64
Phenolics	39.0	0.01	1.00	0.0003	0.85	0.69	0.0429	86.37	1.00	0.0002	0.72	0.53
